# Sensing Performance and Mechanical Properties of Buckypaper Impregnated with Epoxy Resin

**DOI:** 10.3390/nano10112258

**Published:** 2020-11-14

**Authors:** Shiuh-Chuan Her, Wei-Chun Hsu

**Affiliations:** Department of Mechanical Engineering, Yuan Ze University, Chung-Li 320, Taiwan; s1045026@mail.yzu.edu.tw

**Keywords:** buckypaper composite, piezoresistive effect, strain sensitivity, temperature sensitivity

## Abstract

Buckypaper consisting of a carbon nanotube (CNT) sheet has a great potential for sensing and structural applications due to the exceptional piezoresistive and mechanical properties of CNTs. In this work, buckypaper was impregnated with the epoxy resin to improve the fragility and handling capability. The mechanical properties of the buckypaper/epoxy composite were determined by the tensile and nanoindentation tests. A thermogravimetric analyzer (TGA) was used to evaluate the thermal stability. Strain and temperature sensing performances of the buckypaper/epoxy composite based on the piezoresistive effect were investigated using a meter source. Experimental results indicated that the elastic modulus and ultimate strength of the buckypaper/epoxy composite were increased by 82% and 194%, respectively, in comparison with the pristine buckypaper, while the strain and temperature sensitivities were decreased by 33% and 0.2%, respectively. A significant increase of the tensile strength accompanied with a moderate decrease of the strain sensitivity demonstrates that the overall performance of buckypaper/epoxy composite is better than that of pristine buckypaper.

## 1. Introduction

Carbon nanotubes (CNTs) discovered by Iijima [1] with exceptional mechanical, thermal, and electrical properties have attracted wide attention for the development of nanomaterials in recent years. CNTs are considered as ideal reinforcements that can be embedded in a matrix to incorporate the inherent properties to the composites. The use of CNT composite is becoming widespread and has led to different applications. To take advantage of the excellent mechanical properties of CNTs, Gojny et al. [2] have added 0.1 wt% of CNTs as nanofillers in epoxy with enhanced strength and toughness. Utilizing the high flexibility, excellent electrical conductivity of multi-walled carbon nanotube (MWCNT), Aloui et al. [3] developed a flexible transparent conducting electrode by vacuum-filtered films of MWCNT networks on polyethylene terephthalate (PET) substrates. It has been proven that the electrical conductivity of CNT can be significantly changed due to the mechanical strain [4]. Hu et al. [5] proposed a flexible tactile sensor with CNTs deposited on the polymer substrate to detect normal and shear forces. Lai et al. [6] developed a novel sensing system which was fabricated by dispersing CNTs and silver particles in a polydimethylsiloxane (PDMS) polymer using the dielectrophoresis technique.

Buckypaper, a paper-like material, consists of CNTs formed by van der Walls forces [7]. Buckypaper is generally prepared by vacuum filtration of a well-dispersed solution of a randomly distributed CNTs. The free-standing CNT film of buckypaper exhibits porous entangled network structure. The porous structure of the buckypaper is appropriate for a wide range of potential applications. Che et al. [8] developed a buckypaper with high electrical conductivity used as an electrode for supercapacitors. Chen et al. [9] prepared a buckypaper using a laser irradiation process to enhance field emission properties for field emission display. Liu et al. [10] deposited metal alloys onto the buckypaper to increase the Seebeck coefficient for thermoelectric applications. Wang et al. [11] investigated the feasibility of buckypaper as a strain sensor. Lu et al. [12] embedded a buckypaper in the glass/epoxy composite laminate to monitor the manufacturing process. Wang et al. [13] employed a buckypaper in Glass Fiber Reinforced Polymer (GFRP) composite to enhance modes I and II interlaminar fracture toughness and monitor the drilling process of the GFRP. Lu et al. [14] used buckypaper to determine the glass transition temperature of glass/epoxy composite laminate. Zangrossi et al. [15] fabricated MWCNT buckypaper composite as self-heating material for ice protection in aircraft structures. Sharma et al. [16] incorporated buckypaper in epoxy matrix to improve the thermomechanical and electrical properties. Datta et al. [17] embedded buckypaper in the interlaminar region of the glass-fiber-reinforced composite laminates to monitor the fatigue crack growth. Kumar et al. [18] interleaved buckypaper between carbon-fiber-reinforced composite laminates to improve electrical conductivity and reduce lightning strike damage.

Buckypaper is a promising material for a variety of applications. However, the free-standing buckypaper suffers a major drawback in terms of mechanical properties, which limit its commercial applications. Efforts have been devoted to develop buckypaper composite with high elastic modulus and tensile strength. Che et al. [8] proposed a new method of co-packaging buckypaper with conducting polymer and thermosetting resin to improve the tensile strength. Asrafi et al. [19] impregnated the buckypaper with epoxy matrix using vacuum infiltration to enhance the Young’s modulus. Li et al. [20] fabricated a strong and tough CNT film/epoxy composite by resin solution impregnation process on chemical vapor deposition growth CNT film, resulting in a tensile strength and toughness of 405 MPa and 122 J/g, respectively. In this work, buckypaper was impregnated with epoxy matrix using vacuum infiltration. The mechanical properties of buckypaper composite were determined by the tensile and nanoindentation tests. A thermogravimetric analyzer (TGA) was used to evaluate the thermal stability. In addition, the effect of epoxy impregnation on the strain and temperature sensitivities of the buckypaper was investigated.

## 2. Experimental

### 2.1. Preparation of Buckypaper

**Figure 1:**
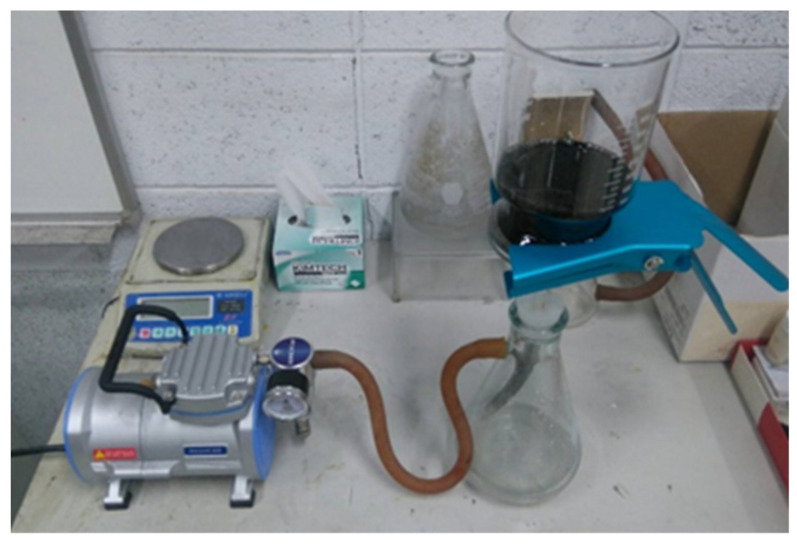
Vacuum filtration setup.

In this work, multi-walled carbon nanotubes (MWCNTs) purchased from Conjutek Co., New Taipei City, Taiwan were used as received. The diameter and length of MWCNTs are in the range of 10–20 nm and 100–200 μm, respectively, while the purity is greater than 98.5%. An amount of 50 mg of MWCNTs were dispersed in an aqueous solution of 250 mL. In addition, a surfactant Triton X-100 of 5 g was added into the solution to separate the MWCNTs bundles into individual MWCNTs. The suspension was dispersed using a sonicator (Q700, Qsonica L.L.C., Newtown, CT, USA). The sonication process was operated at pulse mode with 10 s on and 20 s off for 2 h. Once the sonication process was completed, the suspension was filtered through a PVDF (Polyvinylidene Fluoride) microporous membrane with pore size 0.45 μm and diameter 47 mm. The experimental setup of the vacuum filtration is shown in Figure 1. After filtration, the wet buckypaper was carefully removed from the membrane and submerged in the isopropyl alcohol for 4 h to clean the residual surfactant. Then, the pristine buckypaper was washed by a large amount of distilled water and dried in a thermal chamber overnight at a temperature of 40 °C.

### 2.2. Buckypaper Impregnated with Epoxy

**Figure 2:**
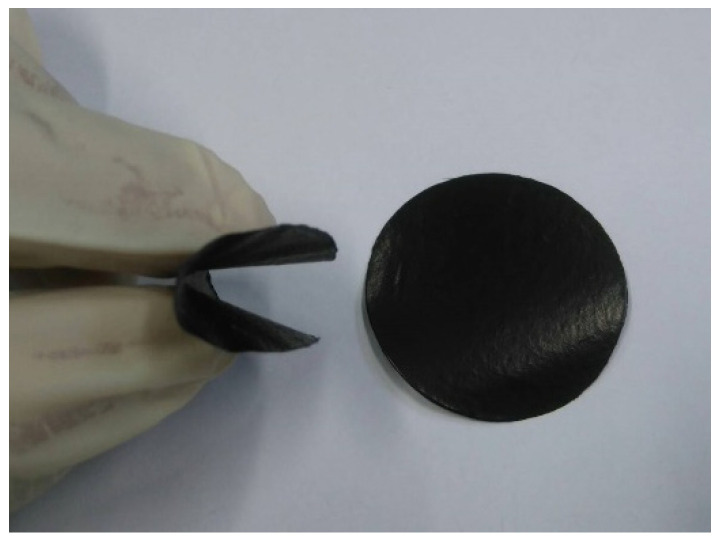
Buckypaper/epoxy composite.

The pristine buckypaper was impregnated with epoxy using a vacuum infiltration technique. The low viscosity epoxy Mungo 4200A part A and curing agent 4200B part B provided by Uchess Co. (New Taipei City, Taiwan) were used. The weight ratio between the epoxy and curing agent was 2:1 as recommended by the manufacturer. The curing agent was added into the liquid epoxy, and slowly stirred for 10 min. Then, the mixture was degassed in a vacuum chamber at room temperature for 15 min to remove trapped air induced by the stirring. Consequently, epoxy resin was poured onto the buckypaper and placed in a vacuum chamber. A vacuum pressure of 0.1 atm was applied to infiltrate the buckypaper with the epoxy resin for 15 min. After that, the buckypaper/epoxy composite was placed between two steel plate and a force equivalent to a pressure of 20 KPa was applied to the system for 4 h. The buckypaper/epoxy composite was then cured in situ at room temperature for 24 h. A typical free standing and flexible buckypaper/epoxy composite is shown in Figure 2 with the diameter and thickness of 36 mm and 0.2 mm, respectively. It can be observed that the buckypaper/epoxy composite exhibits good strength and flexibility to be handled like a traditional mat.

### 2.3. Characterization

The surface morphology and tensile fracture of the pristine buckypaper and buckypaper/epoxy composite were observed using a field emission scanning electron microscopy (JSM 7600F, Jeol Co., Tokyo, Japan) conducted with the accelerated voltage of 10 kV. The thermal stability of the pristine buckypaper and buckypaper/epoxy composite were examined by a thermogravimetric analyzer (TGA) (2-HT TGA, Mettler Toledo, Columbus, OH, USA) under nitrogen atmosphere from room temperature to 800 °C with a heating rate of 10 °C/min. The mechanical properties of the pristine buckypaper and buckypaper/epoxy composite including the elastic modulus, ultimate strength, and fracture strain were determined by a tensile test while the hardness was evaluated through the nanoindentation test. The tensile tests were performed on an universal testing machine equipped with a load cell of 200 N. Rectangular specimens with dimensions of length 30 mm and width 10 mm were prepared and loaded at a constant speed of 0.5 mm/min. Nanoindentation tests were conducted using a nano indenter (Nano Test|Micro Materials Ltd., Wrexham, UK) equipped with a Berkovich diamond indenter to measure the hardness of the pristine buckypaper and buckypaper/epoxy composite.

The fabricated pristine buckypaper and buckypaper/epoxy composite were cut in a rectangular strip with length 30 mm and width 10 mm. The prepared buckypaper sensor was attached on an aluminum test specimen. The aluminum specimen was subjected to a four-point bending test. The resistance changes of the pristine buckypaper and buckypaper/epoxy composite sensors in response to the applied strain of the four-point bending test were measured by a source meter (Keithley 2450, Beaverton, OR, USA). The gauge factor (GF) defined as the ratio between the relative resistance change and applied strain is used to characterize the strain sensitivity as followed.

(1) $$\mathrm{GF}=\frac{\Delta R}{R_{0}\varepsilon }$$

where $R_{0}$ is the initial resistance at zero strain, ΔR represents the resistance change due to the applied strain ε.

To determine the temperature sensitivity, the pristine buckypaper and buckypaper/epoxy composite sensors were placed in a thermal chamber. The temperature was increased from room temperature to 100 °C at a step of 5 °C. A K-type thermocouple was employed next to the sensor to record the temperature. The resistance changes of the pristine buckypaper and buckypaper/epoxy composite sensors were measured by a source meter (Keithley 2450). Temperature coefficient of resistance (TCR) defined as follow is used to evaluate the temperature sensitivity.

(2) $$\mathrm{TCR}=\frac{\Delta R}{R_{0}\Delta T}$$

where $R_{0\;}$is the initial resistance at room temperature, ΔR denotes the resistance change due to the temperature change ΔT.

## 3. Results and Discussions

### 3.1. Surface and Cross-Section Morphologies

**Figure 3:**
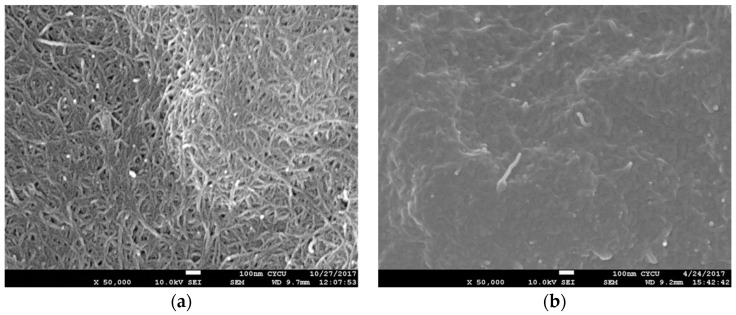
Surface morphology (**a**) pristine buckypaper (**b**) buckypaper/epoxy composite.

**Figure 4:**
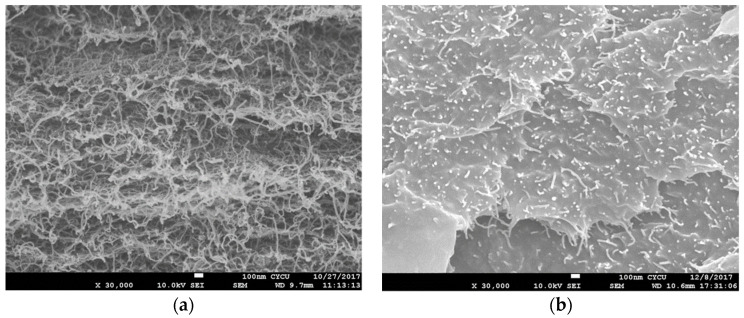
Cross-section view (**a**) pristine buckypaper (**b**) buckypaper/epoxy composite.

The SEM images of surface morphologies of the pristine buckypaper and buckypaper/epoxy composite are shown in Figure 3. It can be seen that an entangled MWCNT network exhibits in the pristine buckypaper due to the van der Waal forces. A dense pack with randomly distributed MWCNTs of the pristine buckypaper indicates a good dispersion of MWCNTs in the suspension. While the MWCNT network of the buckypaper/epoxy composite is invisible since it is covered by the epoxy resin. The SEM images of the fracture surface of the tensile specimens were examined to evaluate the cross-section view of the pristine buckypaper and buckypaper/epoxy composite as shown in Figure 4. A layered structure was found in the pristine buckypaper. It demonstrates a high degree of entanglement among the MWCNTs. The cross-section view of the buckypaper/epoxy composite shows a uniform epoxy background surrounding the MWCNTs. It reveals a good impregnation of the epoxy resin in the buckypaper.

### 3.2. Thermal Stability

**Figure 5:**
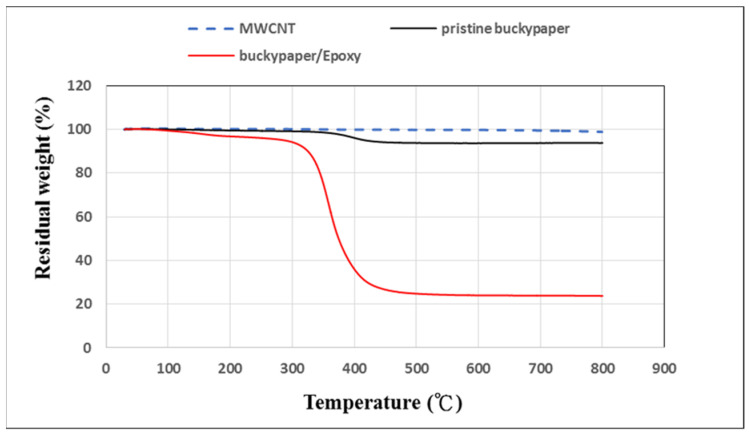
Residual weights of multi-walled carbon nanotubes (MWCNTs), pristine buckypaper, and buckypaper/epoxy composite in the TGA tests.

To evaluate the impurity and epoxy resin, TGA tests were conducted on the MWCNTs, pristine buckypaper, and buckypaper/epoxy composite using a thermogravimetric analyzer (2-HT TGA, Mettler Toledo, Columbus, Ohio, USA). TGA tests were operated in the nitrogen environment and carried out from room temperature to 800°C with a heating rate of 10°C/min. Figure 5 shows the residual weights of the MWCNTs, pristine buckypaper, and buckypaper/epoxy composite as the temperature increased from room temperature to 800 °C. Both the MWCNTs and pristine buckypaper exhibit a good thermal stability. The residual weights of the MWCNTs, pristine buckypaper, and buckypaper/epoxy composite at temperature of 800 °C were 98.7 wt%, 93.8 wt%, and 23.8 wt%, respectively. It can be observed that the impurities of the MWCNTs and pristine buckypaper are less than 3% and 7%, respectively. Most of the weight loss of the pristine buckypaper can be attributed to the residual surfactant Triton X-100 or moisture. The content of epoxy resin in the buckypaper/epoxy composite is about 76%.

### 3.3. Tensile Properties

**Figure 6:**
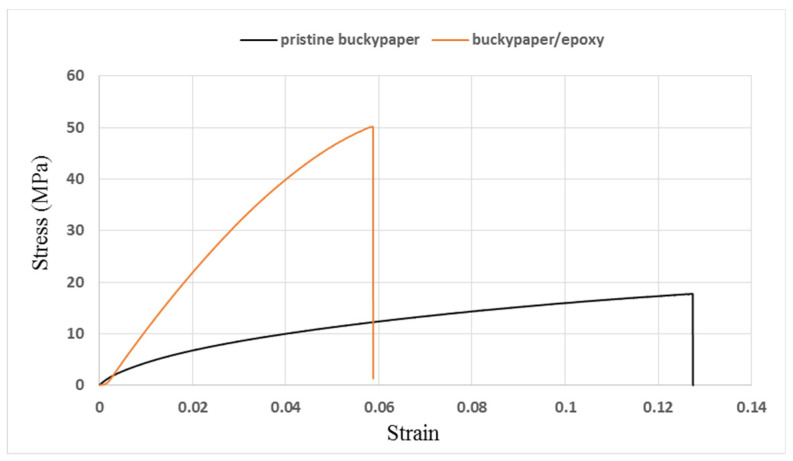
Stress-strain curves of pristine buckypaper and buckypaper/epoxy composite.

The stress vs. strain curves resulted from the tensile tests for the pristine buckypaper and buckypaper/epoxy composite are plotted in Figure 6. The tensile properties such as the elastic modulus, ultimate strength, and fracture strain can be obtained from the stress-strain curve. Three specimens were fabricated and tested for the pristine buckypaper and buckypaper/epoxy composite. The average and standard deviation of the tensile properties for the pristine buckypaper and buckypaper/epoxy composite are presented in Table 1. The tensile properties of the neat epoxy are also listed in Table 1 for the reference. It can be seen that the elastic modulus and ultimate strength of the buckypaper infiltrated with the epoxy are increased by 82.6% and 194%, respectively, in comparison with the pristine buckypaper. For the pristine buckypaper, the interconnection between the MWCNTs is based on the weak van der Waal forces. The loose bond of the entangled MWCNTs network results in a lower elastic modulus and ultimate strength. However, as the buckypaper infiltrated with the epoxy resin, MWCNTs are surrounded by the epoxy resin as shown in Figure 4b. Epoxy infiltrated into the buckypaper acts as the adhesive among the MWCNTs. MWCNTs bonded by the epoxy is superior to the van der Waal force. Thus, the tensile properties of the buckypaper can be improved by infiltration with the epoxy resin. The strong interaction between the MWCNTs and epoxy increases the load transfer, leading to a significantly improvement of both the elastic modulus and ultimate strength.

**Figure 7:**
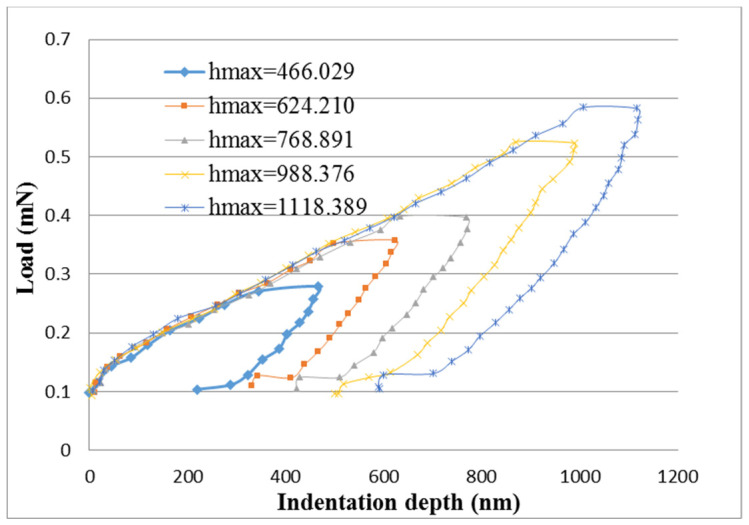
Load vs. indentation depth for the pristine buckypaper in the nanoindentation test.

**Figure 8:**
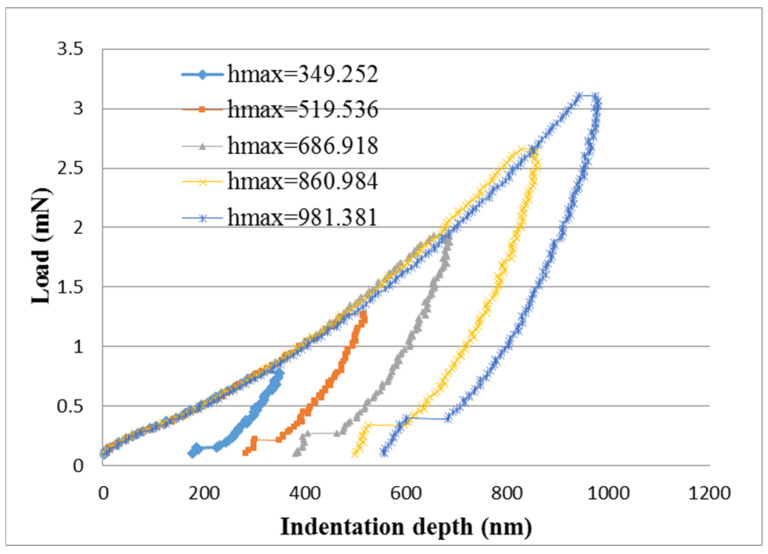
Load vs. indentation depth for buckypaper/epoxy composite in the nanoindentation test.

**Figure 9:**
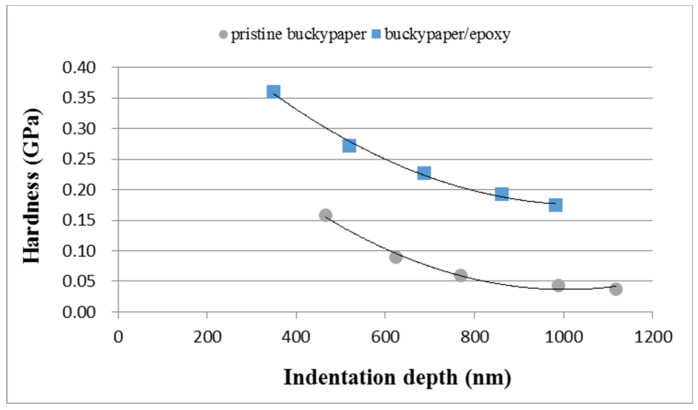
Hardness of the pristine buckypaper and buckypaper/epoxy composite varying with the indentation depth.

In the nanoindentation tests, the indentation depth was increased from 300 nm to 1000 nm. Figure 7 and Figure 8 plot the curves of load versus indentation depth for the pristine buckypaper and buckypaper/epoxy composite, respectively. The hardness of the pristine buckypaper and buckypaper/epoxy composite can be determined from the curve of load vs. indentation using the expression derived by Oliver and Pharr [21]. Figure 9 shows the hardness of the pristine buckypaper and buckypaper/epoxy composite varying with the indentation depth. It appears that the hardness is decreasing with the increase of the indentation depth. In the nanoindentation test, the indentation depth is increasing with the increase of the indentation load. The plastic deformation induced by the indentation is greatly dependent on the level of the indentation load. The accumulated plastic zone of the buckypaper is increasing as the indentation depth increases due to a large indentation load, resulting in a decrease of the hardness. The hardness of the buckypaper/epoxy composite is larger than that of the pristine buckypaper.

### 3.4. Strain Sensing

**Figure 10:**
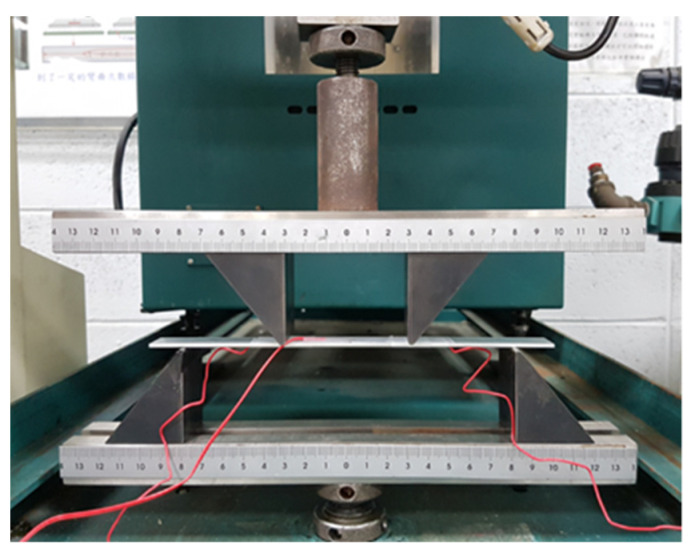
Experimental setup of the four-point bending test.

**Figure 11:**
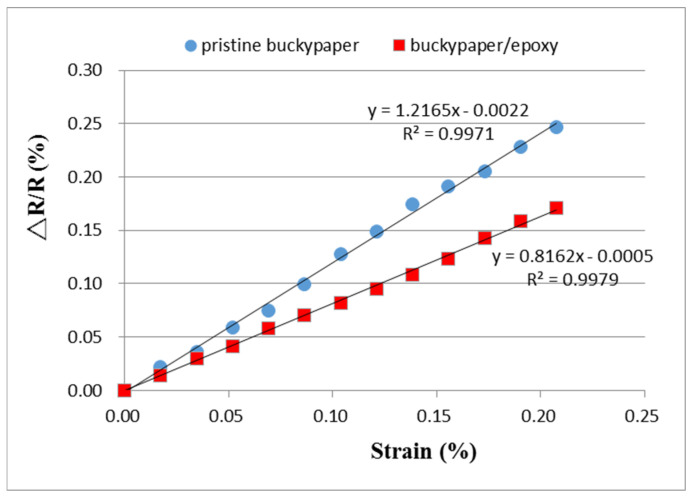
Resistance changes of the pristine buckypaper and buckypaper/epoxy composite varying with the applied strain.

The piezoresistive property of the buckypaper/epoxy composite was investigated by a source meter (Keithley 2450, Beaverton, OR, USA). The buckypaper/epoxy composite with dimensions of 30 × 10${\;\mathrm{mm}}^{2}$ was adhered to the center of an aluminum beam with dimensions of 200 × 20 × 2 ${\mathrm{mm}}^{3}$. The Al specimen was under a four-point bending test using an universal machine (10 KS, Hounsfield, UK) at a loading rate of 5 mm/min as shown in Figure 10. A strain gauge bonded next to the buckypaper/epoxy composite sensor was employed to measure the strain. The buckypaper/epoxy composite and the strain gauge were adhered to the tensile surface of the beam during the flexural test. The electrical resistance of the buckypaper/epoxy composite sensor was determined by a source meter. The electrical resistance changes of the pristine buckypaper and buckypaper/epoxy composite as a function of applied strain were recorded and illustrated in Figure 11. A good linear relationship between the electrical resistance change and applied strain with the coefficient of determination greater than 0.99 demonstrates the feasibility of the buckypaper/epoxy composite as a strain sensor. The gauge factor defined in Equation (1) can be extracted from the slope of the linear curve. The gauge factors for the pristine buckypaper and buckypaper/epoxy composite are 1.22 and 0.82, respectively. The resistance of the buckypaper consists of intrinsic resistance and contact resistance. The intrinsic resistance is dependent on the MWCNTs types (conducting or semiconducting), aspect ratio, and structural defects, while the contact resistance is dominated by the tunneling effect of MWCNTs. The contact resistance is considerably larger than that of the intrinsic resistance. Thus, the resistance of the buckypaper is mainly dependent on the contact resistance. In the four-point bending test, the buckypaper bonded on the Al beam was stretched, leading to an increase of the contact resistance owing to the increase of the distance between the MWCNTs. A positive response of the piezoresistivity to the applied strain for both the pristine buckypaper and buckypaper/epoxy composite was observed as shown in Figure 11. The resistance exhibits a linear increase with the increase of the applied strain. The strain sensitivity of the pristine buckypaper with gauge factor of 1.22 is higher than that of buckypaper/epoxy composite with gauge factor of 0.82. The interaction between the MWCNTs in the pristine buckypaper mainly relies on the weak van der Waal forces. The loose bond of the entangled MWCNTs network makes the conductive pathway of the pristine buckypaper easy to be destroyed by the applied strain. However, as the buckypaper impregnated with the epoxy, the movement of the MWCNTs is constrained by the surrounding epoxy reign. Thus, the conductivity of the buckypaper/epoxy composite is less sensitive to the applied strain in comparison with the pristine buckypaper. A significant increase of the tensile strength accompanied with a moderate decrease of the piezoresistive effect demonstrates that there is a tradeoff between the tensile and piezoresistive properties as the buckypaper infiltrated with the epoxy resin.

### 3.5. Temperature Sensing

**Figure 12:**
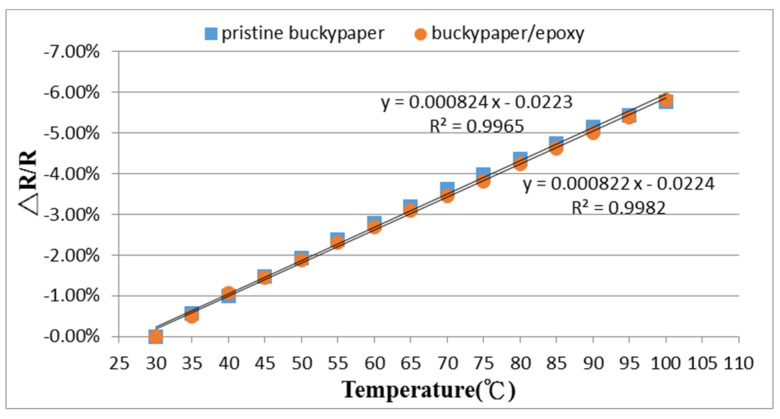
Resistance changes of the pristine buckypaper and buckypaper/epoxy composite varying with the temperature.

The pristine buckypaper and buckypaper/epoxy composite were cut into rectangular strip with dimensions of 30 mm × 10 mm and placed in a vacuum oven. To investigate the temperature sensitivity of the pristine buckypaper and buckypaper/epoxy composite, the temperature was varied from room temperature to 100 °C. The resistance changes in response to the temperature change for both the pristine buckypaper and buckypaper/epoxy composite were recorded as shown in Figure 12. It appears that the resistance is decreasing as the temperature increasing for both the pristine buckypaper and buckypaper/epoxy composite. A negative piezoresistivity was observed for both the pristine buckypaper and buckypaper/epoxy composite. It is well-known that CNTs can exhibit either in a metallic or a semiconducting behavior dependent on its chirality. For the metallic type of CNT, the temperature dependence of the resistance is affected by charge carrier scattering. The probability of carrier scattering is increased as the temperature increases, which causes a decrease in mobility of the charge carriers and an increase in resistance. For the semiconducting type of CNT, the temperature dependence of the resistance is determined by its thermally activated charge carriers. As the temperature increases, the mobility of the charge carriers increases, and thus, the resistance decreases. The negative piezoresistivity of the buckypaper is a result of semiconductive charge transport behavior. Two mechanisms have been proposed by Zhang et al. [22], that is, variable-range hopping and tunnel conduction. Luo and Liu [23] reported that the charge transport in CNT thin films is dictated by a variable-range hopping mechanism, which facilitates the charge carrier mobility and reduces the resistance at high temperature. Gong et al. [24] indicated that the main mechanism for electrical property of the CNT/polymer composite is thermally assisted tunneling on CNT junctions. A higher temperature condition provides more energy for electrons to tunnel through the local potential barrier, which is called thermally assisted tunneling. A good linear relationship between the resistance change and temperature change was observed. The temperature coefficient of resistance (TCR) defined in Equation (2) can be extracted from the slope of the curve. The TCR of the pristine buckypaper and buckypaper/epoxy composite are $-8.24\times 10^{-2}\;{}^{\circ}C^{-1}$ and $-8.22\times 10^{-2}\;{}^{\circ}C^{-1}$, respectively.

## 4. Conclusions

In this work, the pristine buckypaper was infiltrated with epoxy resin. The effect of epoxy resin on the mechanical properties and sensing performance was investigated. The cross-section morphology examined by the field emission scanning electron microscopy illustrated that the epoxy resin was successfully infiltrated through the buckypaper. MWCNTs were uniformly distributed and surrounded by the epoxy resin. The mechanical properties of the buckypaper/epoxy composite determined by the tensile tests show that the elastic modulus and tensile strength are increased by 82.6% and 194%, respectively, in comparison with the pristine buckypaper. The improvement of tensile properties can be attributed to a strong interaction between the MWCNTs and epoxy, leading to a better load transfer. The sensing capability of the buckypaper/epoxy composite based on the piezoresistive effect was investigated using a source meter. It was found that the buckypaper/epoxy composite exhibits a positive piezoresistive behavior to the applied strain and a negative piezoresistive response to the temperature change. The gauge factor used to characterize the strain sensitivity of the buckypaper/epoxy composite is decreased by 32.8% in comparison with the pristine buckypaper. However, the temperature sensitivity of the buckypaper was not affected by the infiltration of the epoxy resin. A good linearity and stability of the piezoresistive response illustrate a great feasibility of the buckypaper/epoxy composite in the applications of strain and temperature sensing.

## Figures and Tables

**Table 1:** Tensile properties of the pristine buckypaper, buckypaper/epoxy composite, and epoxy.

Tensile Properties	Pristine Buckypaper	Buckypaper/Epoxy Composite	Epoxy
Elastic modulus GPa	0.68 ± 0.025	1.24 ± 0.029	0.99 ± 0.01
Ultimate strength MPa	17.3 ± 0.29	50.8 ± 1.60	40.4 ± 0.23
Fracture strain	0.122 ± 0.003	0.060 ± 0.002	0.041 ± 0.0002
